# Long Noncoding RNA ENSG00000254693 Promotes Diabetic Kidney Disease via Interacting with HuR

**DOI:** 10.1155/2022/8679548

**Published:** 2022-04-19

**Authors:** Qun Yu, Jiangong Lin, Qiqi Ma, Yanmei Li, Qianhui Wang, Huimin Chen, Yue Liu, Bing Liu

**Affiliations:** ^1^Department of Nephrology, Shandong Provincial Hospital, Shandong University, Jinan, 250021 Shandong, China; ^2^Department of Nephrology, Shandong Provincial Hospital Affiliated to Shandong First Medical University, Jinan, 250021 Shandong, China

## Abstract

Diabetic kidney disease (DKD) is one of the most common complications of diabetes mellitus (DM), without suitable therapies, causing end-stage renal diseases (ESRDs) ultimately. Moreover, there is increasing evidence demonstrating that long noncoding RNAs (lncRNAs) play crucial roles in the development of DKD. Our RNA sequencing data revealed a large group of differentially expressed lncRNAs in renal tissues of DKD, of which lncRNA ENSG00000254693 (lncRNA 254693 for short) changed drastically. In this study, we found that the expression of lncRNA 254693 was increased in both DKD patients and high-glucose-induced human podocytes. 5′/3′RACE and Northern blot assays were used to find the full length of lncRNA ENSG00000254693 which is 558 nucleotides and nonisoform that existed in human podocyte. Downregulation of lncRNA 254693 remarkably reversed the elevation of inflammation, apoptosis, and podocyte injury caused by high glucose. Then, we did bioinformatics analysis via RBPDB and found that lncRNA 254693 can combine with HuR, a RNA binding protein. Meanwhile, immunofluorescence and in situ hybridization double staining was used to prove the existence of colocalization between them. Intriguingly, lncRNA 254693 knockdown decreased HuR levels, while HuR knockdown also decreased the level of lncRNA 254693 and its stability. After this, RNA immunoprecipitation assay results confirmed the binding association between them again. In addition, we found that HuR was increased in high glucose-induced podocytes, and the silence of HuR could alleviate podocyte injury, inflammation, and apoptosis. These results together suggested a novel feedback regulation between lncRNA 254693 and HuR which could involve in podocyte injury and may serve as a predicted target for DKD therapies.

## 1. Introduction

In recent decades, the number of diabetics has increased dramatically, with an estimated 463 million people living with diabetes in 2019 and expected to rise to 578 million by 2030 (International Diabetes Federation 2019). The incidence and number of DKD patients have also increased significantly, which occurs in 20-40% of diabetic patients, threatening human health and social development seriously [1]. Meanwhile, increasing evidences have demonstrated that long noncoding RNAs (lncRNAs) are involved in the development and progression of some diseases, such as cardiovascular disease, prostate cancer, fatty liver, DM, and DKD [2–5]. lncRNAs are defined as noncoding transcripts over 200 nucleotides in length without protein coding capacity, regulating the expression of target genes [6]. Recently, some studies have revealed that diverse lncRNAs regulate the development of DKD via stimulating renal inflammation [7], accelerating renal interstitial fibrosis [8], and promoting proliferation, epithelial-mesenchymal transition [9], and so on. However, the role of lncRNA 254693 in the pathological process of DKD remains largely unknown.

Recent studies have found that lncRNAs mediate DKD by interaction with proteins and the abundance of proteins is modulated by lncRNAs [10, 11]. Bioinformatics analysis found lncRNA 254693 could bind with human antigen R (HuR). HuR, a member of the embryonic lethal abnormal vision (ELAV) family of RNA-binding proteins [12], was found to be involved in many diseases, such as hepatocellular cancer and colorectal cancer, by regulating mRNA splicing, transportation, and stability [13, 14]. It was reported that HuR could engage with a variety of RNAs, including coding and noncoding RNA transcripts [15]. HuR could stabilize lnc-Sox5 to regulate carcinogenesis of tongue cancer [16]. Given that we speculate lncRNA 254693 could interact with HuR to regulate podocyte injury and DKD, thus, comprehensive investigations are required.

Podocytes are terminally differentiated epithelial cells that cover the surface of glomerulus and play an important role in maintaining the permeability of the glomerular filtration barrier and achieving selective filtration [17, 18]. Podocyte injury is an early step in the DKD progression, which can be estimated through the expression levels of podocyte-related proteins such as desmin and ZO-1. Furthermore, various studies have outlined that inflammation and apoptosis are two features of podocytes injury and play critical roles in the pathogenesis of DKD. Therefore, the study about the potential mechanisms of lncRNA 254693 in podocyte injury, inflammation, and apoptosis will be useful to find new therapeutic target for the treatment and prevention of DKD.

In this study, we investigated the expression of lncRNA 254693 in DKD patient samples and the expression and function in cultured human podocytes. Meanwhile, lncRNA 254693 was predicted to combine with HuR. As a consequence, we continued to investigate the interplay between lncRNA 254693 and HuR, and their potential functions in DKD development might provide a novel idea for treating DKD.

## 2. Material and Methods

### 2.1. Patients and Samples

Human serum samples were collected from DKD patients in the Department of Nephrology of Shandong Provincial Hospital, and the serum samples in the normal control group were obtained from Physical Examination Center. Informed consent was obtained from every patient. The study was endorsed by the Clinical Research Ethics Committee of Shandong Provincial Hospital.

### 2.2. Cell Culture

The immortalized human podocytes were cultured in RPMI 1640 medium (Gibco, USA) supplemented with 10% fetal bovine serum (Gibco, USA) and 1% penicillin-streptomycin under permissive conditions 33°C with 5% CO_2_ in a humidified atmosphere during the proliferation phase and next cultured in a 37°C with 5% CO_2_ incubator for 7-14 days to induce differentiation. Then, HPCs could be used in related experiments. According to the different conditions, HPCs were cultured with medium containing low glucose (LG group, 5.5 mM D-glucose), high glucose (HG, 30 mM D-glucose), and hyperosmolality (HO, 5.5 mM D-glucose plus 24.5 mM mannitol).

### 2.3. Cell Transfection

Transfection of podocytes was performed (1 × 10^6^ cells/well in a 6-well plate) with the indicated siRNAs using Lipofectamine3000 reagent (Invitrogen, USA) for 8-12 h. After HPCs were treated with HG for 24-36 h, target siRNA or NC siRNA was transfected into them. Cells that did not need to be transfected siRNA were treated with LG as the control. lncRNA ENSG00000254693 siRNA (5′-GGCAGAAGCUUCACUUUAU-3′), HuR siRNA (5′-GCUCAGAGGUGAUCAAAGATT-3′), and NC siRNA (a negative control) were purchased from Genomeditech.

### 2.4. Fluorescence in Site Hybridization (FISH)

Ribo™ lncRNA FISH Probe Mix (Red) (RiboBio, China) was utilized according to the standard protocol. Cover glasses were placed, and the podocytes were inoculated into 6-well plates under different conditions until the cell density reached approximately 70%. Podocytes were fixed in 4% paraformaldehyde for 10 min, followed by 0.5% Triton X-100 for 5 min, and then blocked with prehybridization buffer for 30 min at 37°C. Discarding the prehybridization buffer, hybridization buffer containing the probe was added, then incubated at 37°C overnight. After washing with SSC buffer, podocytes were added DAPI to stain the nucleus. Images were observed and captured under a fluorescence microscope (Leica, Germany).

### 2.5. Quantitative Real-Time PCR (qRT-PCR) Analysis

The total RNAs in podocytes under different conditions were extracted using TRIzol reagent (Invitrogen, CA, USA). Total RNAs in blood samples from patients were extracted using the BIOG cfRNA Easy Kit (BIOG, China). A NanoDrop 2000 spectrophotometer (Thermo Fisher Scientific, USA) was used to detect the concentration and purity of the extracted total RNA. Then, 1000 ng RNA from each sample was reverse transcribed into complementary DNA (cDNA) using the PrimeScript® RT Reagent Kit with gDNA Eraser (Takara, China). The primers of target genes were designed and synthesized by Biosune (Shanghai, China), and the sequences of them are listed in Table 1. PCR amplification was performed using SYBR® Premix Ex Taq (Takara, China) according to the LightCycler® 480 Real-Time PCR system (Roche, USA). *β*-Actin was used as an internal reference gene, and the relative quantity of target genes was calculated by the 2^−∆∆CT^ method.

### 2.6. Western Blot Analysis

The total protein was extracted from podocytes by RIPA lysis buffer (Beyotime, China) containing proteinase inhibitors and phosphatase inhibitors (Solarbio, China). The equal protein was separated by SDS-PAGE and transferred to PVDF membranes (Millipore, USA). The membranes were blocked with 5% nonfat milk for 1-2 h at room temperature and incubated overnight at 4°C with the following primary antibody against anti-desmin (1 : 5000, 16520-1-AP, Proteintech, China), anti-ZO-1 (1 : 1000, PA5-85256, Invitrogen, USA), anti-IL-6 (1 : 1000, 66146-1-Ig, Proteintech, China), anti-TNF-*α* (1 : 1000, 60291-1-Ig, Proteintech, China), anti-cleaved-caspase-3 (1 : 1000, 19677-1-AP, Proteintech, China), and anti-HuR (1 : 100, 11910-1-AP, Proteintech, China). After washing three times with TBST for 5 min each time, the membranes were incubated with horseradish peroxidase- (HRP-) conjugated goat anti-rabbit IgG (1 : 5000, SA00001-2, Proteintech, China) and horseradish peroxidase-conjugated goat anti-mice IgG (1 : 5000, SA00001-1, Proteintech, China) at room temperature for 1 h, respectively. The protein was exposed with ECL reagent (Millipore, USA) and Amersham Imager 680 (GE, USA) and analyzed by ImageJ software. *β*-Actin (1 : 5000, 66009-1-Ig, Proteintech, China) was used as an internal reference.

### 2.7. Immunofluorescence Assay (IF)

Podocytes were plated in 6-well plates under different conditions and then fixed with 4% paraformaldehyde. After penetration with 0.1% Triton X-100, the cells were blocked with 5% BSA for 30 min at room temperature. Next, the cells were incubated at 4°C overnight with primary antibody: anti-desmin (1 : 200, 16520-1-AP, Proteintech, China), anti-ZO-1 (1 : 200, PA5-85256, Invitrogen, USA), and anti-HuR (1 : 100, 11910-1-AP, Proteintech, China). Then, the cells were incubated with a fluorescent secondary antibody: goat anti-rabbit IgG H&L (Alexa Fluor® 488) (1 : 200, ab150077, Abcam, USA) and goat anti-rabbit IgG H&L (Alexa Fluor® 594) (1 : 200, ab150080, Abcam, USA) for 1-2 h at 37°C away from light. After incubation with DAPI for 10 min, images were observed and captured under a fluorescence microscope (Leica, Germany).

### 2.8. Transwell Migration Assays

600 *μ*L RPMI 1640 medium containing 10% FBS was added to the lower chamber of Transwell (Corning, USA); 5 × 10^4^ cells/mL HPC in 200 *μ*L serum-free RPMI 1640 medium were added to the upper chamber. Cells were incubated under 37°C with 5% CO_2_ for 48 h, then fixed with 4% paraformaldehyde for 15 min, penetrated with 0.1% Triton X-100 for 5 min, and the noninvading cells were removed. Next, the invaded cells were stained with hematoxylin and dehydrated using graded ethanol. Finally, cells were observed and captured under a light microscope (Leica, Germany).

### 2.9. RNA Immunoprecipitation (RIP)

RNA immunoprecipitation assays were performed by Magna RIP RNA-Binding Protein Immunoprecipitation Kit (Millipore, USA) according to the manufacturer's instructions. In brief, cells were collected in RIP lysis buffer and centrifuged. A small amount of supernatant was saved as the positive control of input, and the residual supernatants were incubated with anti-HuR antibody or rabbit IgG overnight at 4°C. The related RNA was isolated from the precipitate and then purified. Input RNA and immunoprecipitated RNA were detected by qRT-PCR using the specific primers for lncRNA 254693.

### 2.10. Statistical Analysis

All the experiments were performed three times at least. GraphPad Prism 8 and SPSS 19.0 were used to analyze experimental data and draw statistical graphs. Values were shown as the mean ± s.d. Statistical significance between two groups determined by two tailed and unpaired *t*-test and significance among multiple groups was determined by one-way ANOVA. *P* < 0.05 was considered as statistically significant.

## 3. Results

### 3.1. lncRNA 254693 Is Upregulated in DKD Patient Samples

**Figure 1:**
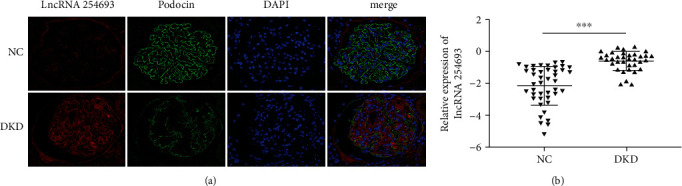
lncRNA 254693 is upregulated in DKD patient samples. (a) FISH staining showing the expression of lncRNA 254693 in the DKD tissues of control (*n* = 10) and DKD patients (*n* = 8). Scale bar = 50 *μ*m. (b) The mRNA expression levels of lncRNA 254693 were detected by qRT-PCR in normal control serums (*n* = 43) and DKD patient serums (*n* = 34). The expression level was shown with the  log_10_2^−Δ*CT*^, Δ*CT* = CT_objective gene_ − CT_*β*−actin_. ^∗∗∗^*P* < 0.001 versus the DKD group. The experiment was repeated three times independently. NC: normal control; DKD: diabetic kidney disease.

It has been reported that changes in the expression of lncRNA are associated with the development of DKD. Therefore, we used RNA sequencing and data analysis to screen differentially expressed lncRNAs in renal tissues of DKD, taking paracarcinoma renal tissues as the control. We selected lncRNA 254693 which was upregulated in DKD as the target of this study. To verify this result, we examined the expression level of lncRNA 254693 in renal tissues and serums of DKD by FISH and qRT-PCR, respectively. The results showed that the expression of lncRNA 254693 was significantly increased in DKD accompanied by the absence of podocin, a podocyte-specific protein (Figure 1(a)). Meanwhile, we found that the expression levels of lncRNA 254693 in DKD serums were higher than those in normal control serums (Figure 1(b)).

### 3.2. Silencing of lncRNA 254693 Inhibits Podocyte Inflammation and Apoptosis

**Figure 2:**
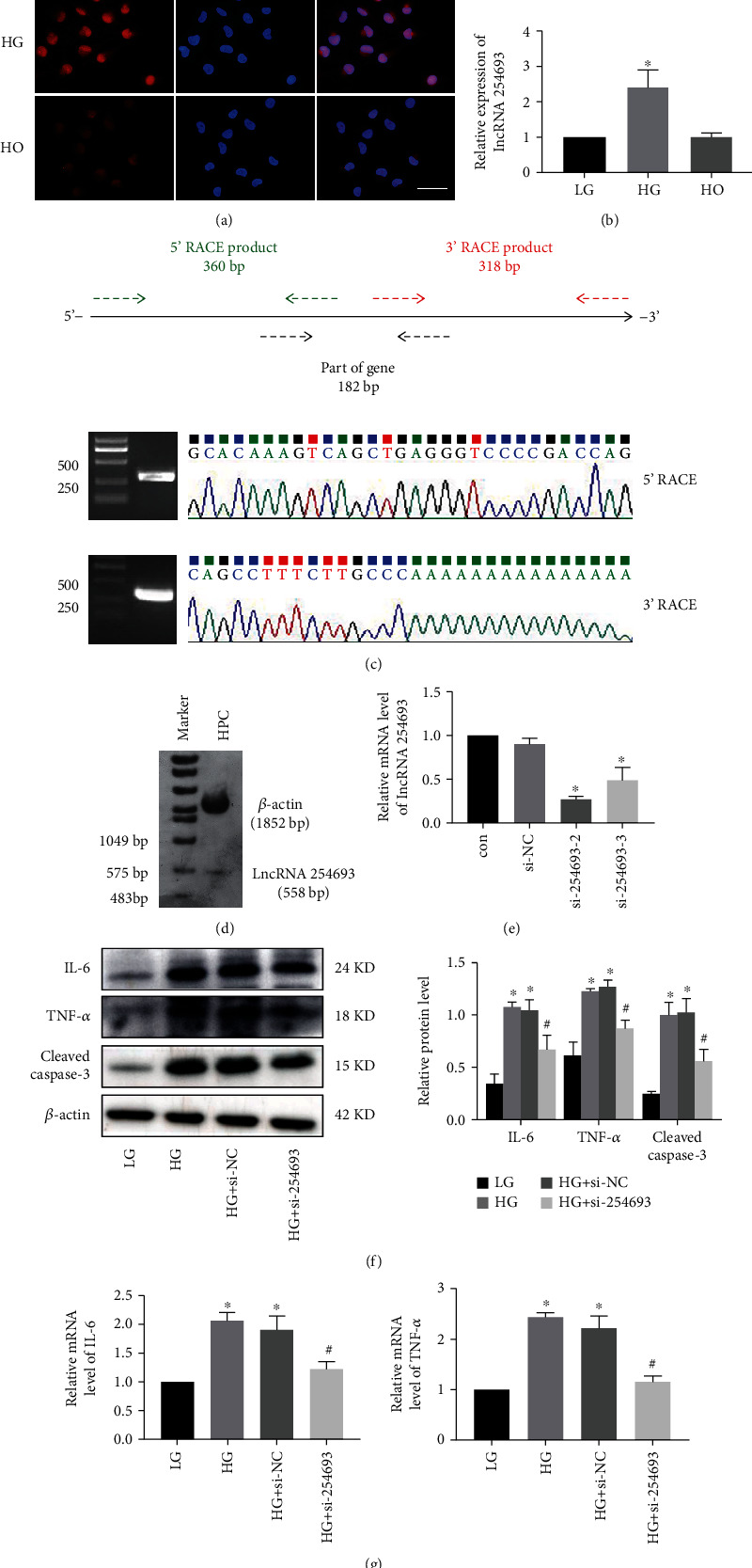
The effect of lncRNA 254693 in podocyte inflammation and apoptosis. (a) The expression of lncRNA 254693 in human podocytes indicated by FISH assay. Scale bar = 50 *μ*m, *n* = 3. (b) The expression of lncRNA 254693 in human podocytes measured by qRT-PCR. ^∗^*P* < 0.05 versus the LG group, *n* = 3. (c) 5′ and 3′ rapid amplification of cDNA end (RACE) assays in human podocytes to detect the whole sequence of lncRNA 254693. (d) Northern blot analysis to confirm the length and expression of lncRNA 254693. (e) The level of lncRNA 254693 after silencing of lncRNA 254693 in podocytes. ^∗^*P* < 0.05 versus the control group, *n* = 3. (f) The protein levels of IL-6, TNF-*α*, and cleaved caspase-3 in response to the treatment of LG, HG, HG+si-NC, and HG+si-254693. ^∗^*P* < 0.05 vs. the LG group, ^#^*P* < 0.05 vs. the HG group, *n* = 3. (g) The mRNA levels of IL-6 and TNF-*α* in response to the treatment of LG, HG, HG+si-NC and HG+si-254693. ^∗^*P* < 0.05 vs. the LG group; ^#^*P* < 0.05 vs. the HG group, *n* = 3. LG: low-glucose; HG: high-glucose; HO: hyperosmolality; NC: negative control.

Podocytes are vital function cells in the development of DKD and cannot regenerate; thus, we focused on podocytes in this study. Initially, the distribution of lncRNA 254693 in podocytes was detected by FISH, indicating that lncRNA 254693 was located in both the nucleus and cytoplasm, but primarily in the nucleus (Figure 2(a)). qRT-PCR was performed to determine the expression of lncRNA 254693, and the results showed that after treatment with high glucose for 48 h, the expression of lncRNA 254693 was increased significantly compared with low glucose (Figure 2(b)). Then, we characterized lncRNA 254693 by 5′/3′ rapid amplification of cDNA end (5′/3′-RACE) experiments in podocytes. The alignment results indicated that the full length of lncRNA 254693 is 588 nucleotides (Figure 2(c)). And Northern blot experiment proved that there was no existence of isoforms of lncRNA 254693 in podocytes (Figure 2(d)).

Inflammation and apoptosis are two key events for podocyte injury. Subsequently, western blot results showed that high-glucose treatment significantly promoted the expression level of proinflammatory cytokines IL-6 and TNF-*α* and apoptosis-related protein cleaved caspase-3 (Figure 2(f)). Subsequently, to explore the function of lncRNA 254693 in high glucose-induced podocytes, we transfected lncRNA 254693 siRNA into podocytes and the knockdown efficiency was shown with statistical difference (Figure 2(e)). lncRNA 254693 knockdown reduced the levels of IL-6, TNF-*α*, cleaved caspase-3 (Figure 2(f)). In agreement with this, qRT-PCR results confirmed the alternations of IL-6 and TNF-*α* expression again (Figure 2(g)). Therefore, these data collectively suggested that lncRNA 254693 positively regulated inflammation and apoptosis in podocytes.

### 3.3. Silencing of lncRNA 254693 Inhibits Podocyte Injury

**Figure 3:**
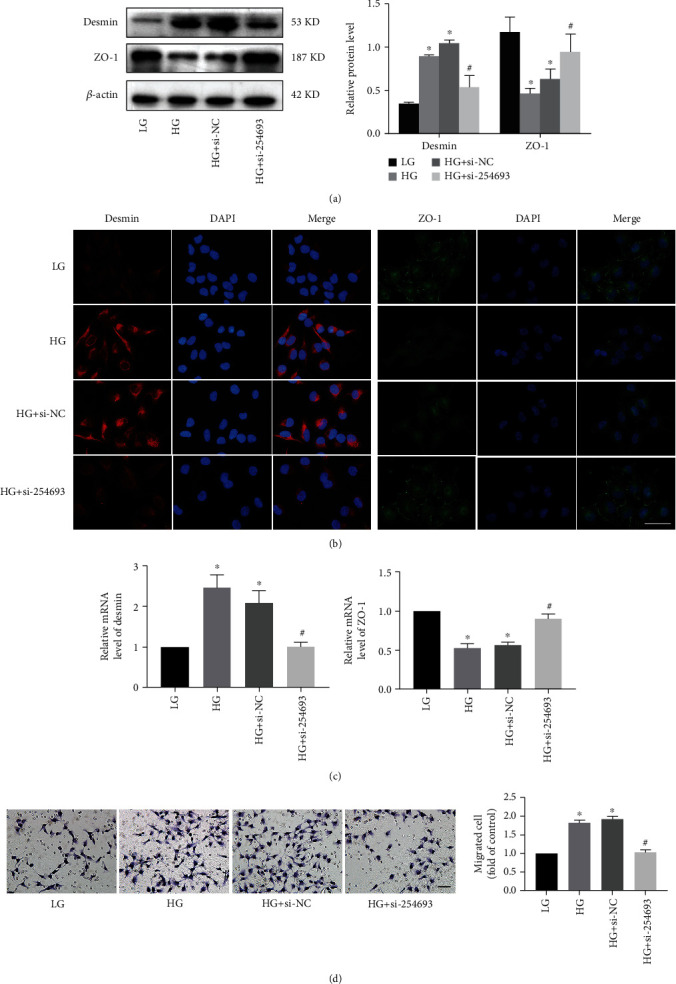
Silencing of lncRNA 254693 inhibits podocyte injury. (a) Western blot analysis of desmin and ZO-1expression in human podocytes with different conditions. ^∗^*P* < 0.05 vs. the LG group, ^#^*P* < 0.05 vs. the HG group, *n* = 3. (b) Representative immunofluorescence images of desmin and ZO-1. Scale bars = 50 *μ*m, *n* = 3. (c) qRT-PCR analysis of desmin and ZO-1 expression in human podocytes with different conditions. ^∗^*P* < 0.05 vs. the LG group; ^#^*P* < 0.05 vs. the HG group, *n* = 3. (d) Representative migration ability of human podocytes. Scale bars = 50 *μ*m. ^∗^*P* < 0.05 vs. the LG group; ^#^*P* < 0.05 vs. the HG group, *n* = 3.

A large number of studies have shown that podocyte injury is an early step in the DKD progression. Based on the above findings, we thus investigated the expression of desmin and ZO-1, two well-known podocyte-typical markers. Firstly, western blot assay and immunofluorescence assay showed that the protein levels of desmin were increased and those of ZO-1 were reduced, while lncRNA 254693 silencing dramatically repressed the expression of desmin and increased the expression of ZO-1 (Figures 3(a) and 3(b)). Meanwhile, qRT-PCR also illustrated that the mRNA expression of desmin was reduced and ZO-1 was increased by knockdown of lncRNA 254693 (Figure 3(c)). In addition, we detected the migration ability of podocytes and observed that knockdown of lncRNA 254693 reversed the migration ability of podocytes, which was significantly enhanced by high glucose (Figure 3(d)). These concluded that lncRNA 254693 silencing inhibited podocyte injury induced by high glucose.

### 3.4. lncRNA 254693 Could Bind with HuR and Regulate the Nucleocytoplasmic Shuttling of HuR

**Figure 4:**
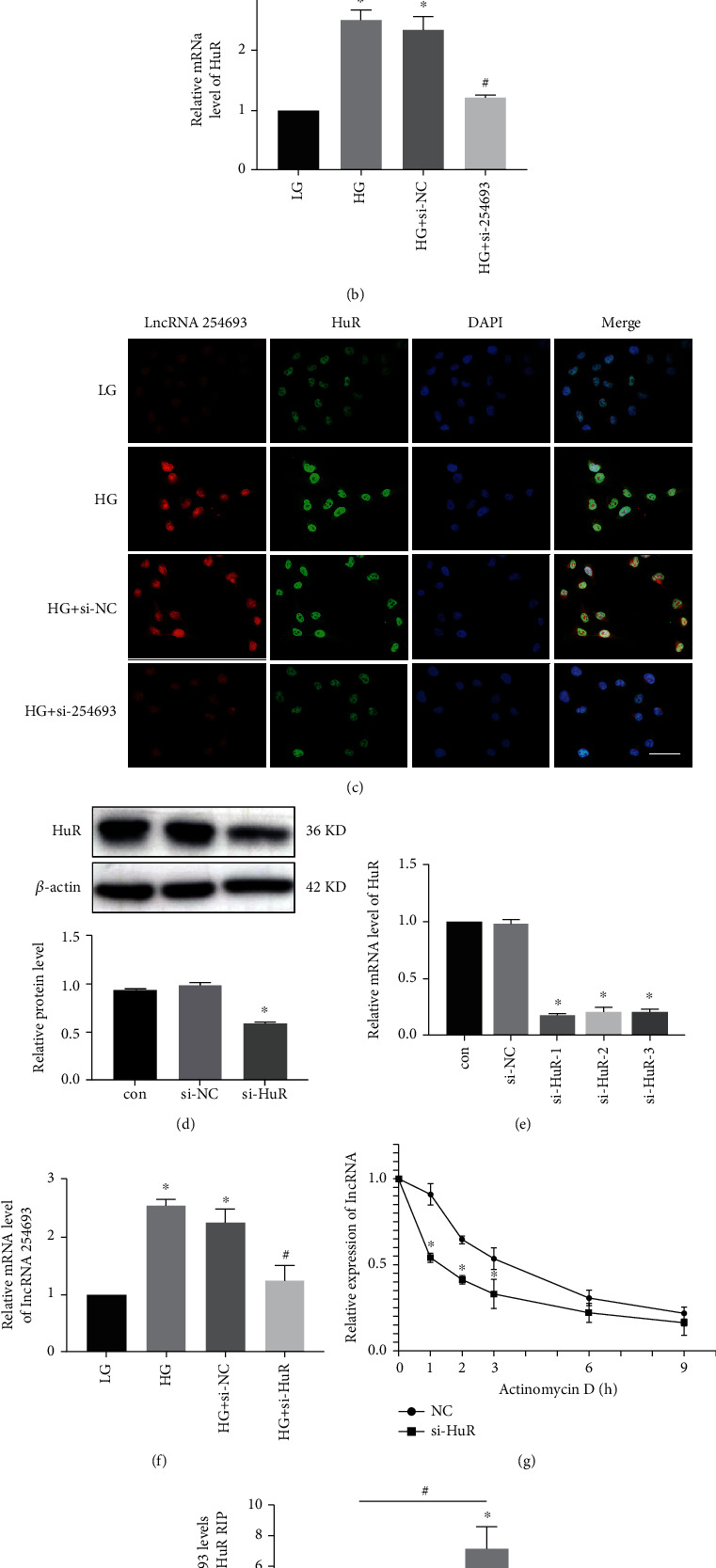
The interaction between lncRNA 254693 and HuR. (a) Western blot assay for the expression of HuR in human podocytes after lncRNA 254693 knockdown, *n* = 3. (b) qRT-PCR for the mRNA level of HuR in human podocytes after lncRNA 254693 knockdown. ^∗^*P* < 0.05 vs. the LG group, ^#^*P* < 0.05 vs. the HG group, *n* = 3. (c) Immunofluorescence and in situ hybridization double staining of human podocytes with different conditions to detect lncRNA 254693 (red) and HuR (green) expression and colocalization. Scale bars = 50 *μ*m, *n* = 3. (d, e) The efficiency of HuR knockdown was confirmed by decreased protein and mRNA expression level. ^∗^*P* < 0.05 vs. the control group, *n* = 3. (f) The expression of lncRNA 254693 in human podocytes after HuR knockdown. ^∗^*P* < 0.05 vs. the LG group; ^#^*P* < 0.05 vs. the HG group, *n* = 3. (g) The effect of HuR on lncRNA 254693 RNA stability. Human podocytes were transfected with HuR siRNA and actinomycin D (5 *μ*g/mL) was then used to treat cells at different time points. RNA was detected by qRT-PCR. ^∗^*P* < 0.05 vs. the NC group, *n* = 3. (h) Relative enrichment of lncRNA 254693 associated with HuR in human podocytes detected by anti-HuR RIP (IgG as negative control). ^∗^*P* < 0.05 vs. IgG, ^#^*P* < 0.05 vs. the HG group.

Generally, lncRNAs work by binding to target genes; thus, we did bioinformatics analysis to find out the lncRNA 254693-related binding proteins. We found that lncRNA 254693 can combine with HuR via RBPDB. Firstly, the expression of HuR was elevated in HG-induced podocytes, and knockdown of lncRNA 254693 markedly repressed the protein and mRNA expression of HuR (Figures 4(a) and 4(b)). Then, according to immunofluorescence and in situ hybridization double staining, we observed that lncRNA814.1 was able to colocalize with HuR and HG promoted HuR shuttle from the nucleus to the cytoplasm, but knockdown of lncRNA 254693 suppressed this process (Figure 4(c)). Subsequently, to further prove that there is an interaction between lncRNA 254693 and HuR, we knocked down HuR in podocytes via transfecting siRNA (Figures 4(d) and 4(e)). Intriguingly, the results showed that downregulated HuR decreased the expression of lncRNA 254693 (Figure 4(f)). Then, podocytes transfected with HuR siRNA were treated with actinomycin D to block RNA synthesis. We found the half-life of lncRNA 254693 in HuR knockdown cells (t1/2 1.5 h) was lower than that in negative control cells (t1/2 3 h) (Figure 4(g)). Furthermore, RNA immunoprecipitation assay was performed in podocytes using HuR antibody to verify the interaction between HuR and lncRNA 254693. lncRNA 254693 was found to be preferentially enriched in HuR antibody captured precipitates compared with IgG control, and it was more obvious in the high-glucose condition (Figure 4(h)).

### 3.5. Downregulation of HuR Inhibited the Injury of Podocytes Induced by HG

**Figure 5:**
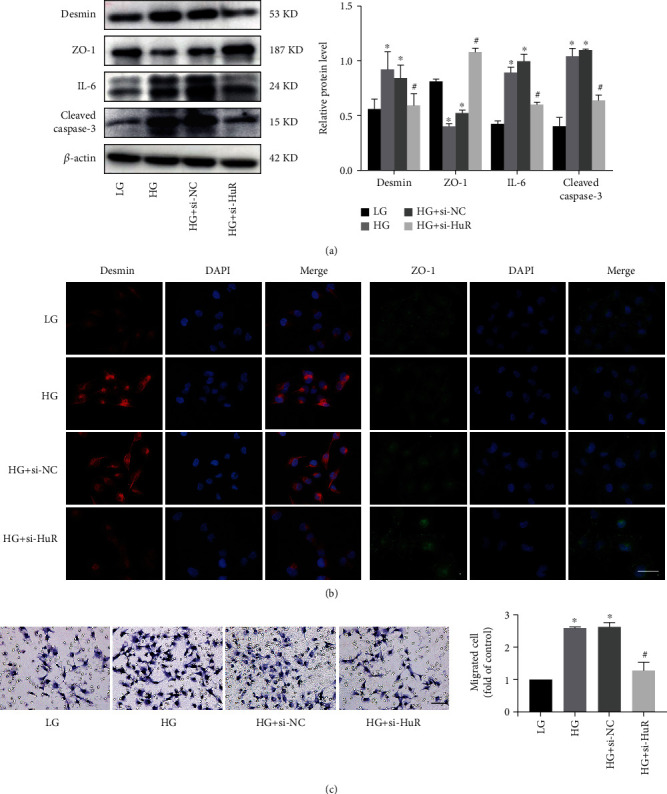
HuR knockdown relieved podocyte injury, inflammation, and apoptosis. (a) The protein level of desmin, ZO-1, IL-6, and cleaved caspase-3 in HuR knockdown human podocytes detected by western blot. ^∗^*P* < 0.05 vs. the LG group; ^#^*P* < 0.05 vs. the HG group, *n* = 3. (b) Representative immunofluorescence images of desmin and ZO-1. Scale bars = 50 *μ*m, *n* = 3. (c) Representative migration ability of podocytes. Scale bars = 50 *μ*m. ^∗^*P* < 0.05 vs. the LG group; ^#^*P* < 0.05 vs. the HG group, *n* = 3.

We further examined the effect of HuR on podocyte injury induced by high glucose and found that the protein level of ZO-1 was enhanced and the levels of desmin, IL-6, and cleaved caspase3 were reduced after HuR knockdown in a high-glucose condition (Figure 5(a)). Consistently, immunofluorescence staining showed the alternations of desmin and ZO-1expression in podocytes (Figure 5(b)). In addition, silence of HuR could decrease the ability of podocyte migration compared with the HG group (Figure 5(c)). Therefore, we had reason to believe that lncRNA 254693 can regulate podocyte injury via the positive feedback with HuR.

## 4. Discussion

Recent evidences suggest that lncRNAs are essential to many biological processes, such as chromatin modification, transcriptional regulation, posttranscriptional regulation of RNA, cell proliferation, differentiation, and apoptosis [19, 20]; which are thought to be a crucial gene regulator by binding to the corresponding mRNAs and play an important role in the pathophysiology of DKD. Given that only few lncRNAs in the DKD have been studied, we used RNA sequencing, a widely used method for transcriptomic analysis, to screen differential expression lncRNAs from transcriptome of DKD renal tissues. Increasing evidence confirmed that RNA sequencing could investigate the functions and biological patterns of lncRNAs, finding candidate targets, and biomarkers for research [21, 22]. Subsequently, we selected lncRNA 254693 with significantly elevated expression in DKD as the research target and the biological function and molecular mechanism of lncRNA 254693 in DKD remain unknown. In our study, we found that compared with the normal control, the expression of lncRNA 254693 was significantly increased in DKD, no matter if it is the tissue or serum of patients, suggesting that lncRNA 254693 participate in the progression of DKD. Subcellular distribution analysis showed that lncRNA 254693 is located in both the nucleus and cytoplasm, which implied a potential of extensive functions in various biological processes.

Podocyte is a critical component of the glomerular filtration barrier which plays a crucial role in the pathogenesis of DKD [23]. Continuous high-glucose stimulation causes the foot process effacement and inflammation, leading to cell detachment, which is a terminal event in podocyte injury, accelerating further glomerular damage and the development of DKD [24, 25]. Existing evidences have suggested that inflammation and apoptosis are the key characteristics, associating with the detachment of podocytes and aggravating their injury [26]. In this study, we found that proinflammatory cytokines IL-6 and TNF-*α* and apoptosis-related protein cleaved caspase-3 were upregulated induced by high glucose. Similarly, Hameed et al. observed that IL-1*β*, IL-6, and TNF-*α* expressions were increased in DKD [27]. Furthermore, it has been reported that the loss of glomerular filtration barrier function in DKD is related to change of the expression levels of podocyte-specific proteins such as podocin, desmin, and ZO-1 [28]. Desmin, a marker of podocyte injury, is an intermediate filament protein among cytoskeletal proteins, which is expressed in the glomerulus when podocyte is injured [29]. ZO-1, a tight junction protein, is negatively correlated with podocyte injury. Consistently, our results showed that compared with the low-glucose control, the level of desmin was increased and the level of ZO-1 was reduced in high-glucose-treated podocytes.

Intriguingly, lncRNA 254693 knockdown reversed IL-6, TNF-*α*, cleaved caspase-3, desmin, and ZO-1 expression changes and reduced the migration ability of high-glucose-treated podocytes. All above indicated that inhibition of lncRNA 254693 has a protective effect in high-glucose-induced inflammation, apoptosis, and podocyte injury and targeting lncRNA 254693 may cause a series of influences in DKD, which may be a novel target for DKD treatment.

Studies have found that lncRNAs can interact with proteins and regulate the expression of them, especially the evidence suggesting that lncRNA-RBP interactions are relevant as protein-protein interactions in the regulation of gene expression [30, 31]. According to report, lncRNA ASB16-AS1 associated with RNA-binding protein HuR and regulated the expression of HuR posttranslationally [15]. lncRNA Airn protected podocytes from DKD conditions via interacting with Igf2bp2, a RNA-binding protein, promoting Igf2 and Lamb2 translation [32]. In our study, bioinformatics analysis results showed that lncRNA 254693 can combine with HuR which can bind transcripts in AU-rich element and stabilizes target mRNAs. Interestingly, we found that the HuR level was downregulated when lncRNA 254693 was inhibited, and the expression and stability of lncRNA 254693 was downregulated when HuR was inhibited. Furthermore, RNA immunoprecipitation assay verified the relationship between lncRNA 254693 and HuR again. Taking our results together, we believed that lncRNA 254693 and HuR can interact with each other and form a feedback loop.

Generally, HuR is mostly localized in the nucleus and its function is often linked with its cellular translocation from the nucleus to the cytoplasm [33]. Therefore, the translocation of HuR from the nucleus to the cytoplasm, which we observed in this study by immunofluorescence and in situ hybridization double staining, is a key step for protecting target mRNAs from degradation [34]. Suzuki et al. found that HuR can modulate mRNA stability of the inflammatory factors (IL-6, TNF-*α*) and apoptosis-related genes [35–37]. Similarly, we observed that IL-6 and cleaved caspased-3 expression levels were downregulated along with the reduction of HuR expression and shuttling after knockdown of HuR. Meanwhile, HuR has been reported to modulate DKD progression [12]. In our study, we found that the expression level of desmin was decreased and ZO-1 was increased when HuR knockdown. Therefore, we speculated that HuR is important for DKD progression, and knockdown of HuR can reduce the damage caused by high-glucose stimulation. Considering the role of lncRNA 254693 and HuR together will bring a new direction for the research of DKD.

## 5. Conclusions

Our study provided evidences to demonstrate that lncRNA 254693 was involved in the progression of DKD and acted as a key regulator of podocyte injury via interacting with HuR. There is a positive feedback loop between lncRNA814.1 and HuR; thus, inhibition of both lncRNA 254693 and HuR could alleviate the inflammation, apoptosis, and podocyte injury induced by high glucose. These findings provide a new insight and therapeutic target for prevention and treatment of DKD.

## Figures and Tables

**Table 1:** Primers for real-time PCR.

Gene	Primer	Sequence
lncRNA 254693	Forward	5′-CTGGATGAGAGGAAATGGGCA-3′
	Reverse	5′-TCTTCCCCCTAGAAACCTCCTC-3′
Desmin	Forward	5′-AGGACCGATTTGCCAGTGAG-3′
	Reverse	5′-CTTGAGGTGCCGGATTTCCT-3′
ZO-1	Forward	5′-CCCCCAACTCAAACCGAAGA-3′
	Reverse	5′-AGATGCTACTTCTGGAGGCTTA-3′
IL-6	Forward	5′-CAATGAGGAGACTTGCCTGGT-3′
	Reverse	5′-GCAGGAACTGGATCAGGACT-3′
TNF-*α*	Forward	5′-GAGGCCAAGCCCTGGTATG-3′
	Reverse	5′-CGGGCCGATTGATCTCAGC-3′
HuR	Forward	5′-TCGGGATAAAGTAGCAGGACAC-3′
	Reverse	5′-AGCGTGTTGATCGCTCTCTC-3′
*β*-Actin	Forward	5′-GAAGACTACGAGCTGCCTGA-3′
	Reverse	5′-CAGACAGCACTGTGTTGGCG-3′

## Data Availability

The data used to support the findings of this study are available from the corresponding author upon request.
